# Pax6 Downregulation Mediates Abnormal Lineage Commitment of the Ocular Surface Epithelium in Aqueous-Deficient Dry Eye Disease

**DOI:** 10.1371/journal.pone.0077286

**Published:** 2013-10-15

**Authors:** Ying Ting Chen, Feeling Y. T. Chen, Trinka Vijmasi, Denise N. Stephens, Marianne Gallup, Nancy A. McNamara

**Affiliations:** 1 Francis I. Proctor Foundation, University of California San Francisco, San Francisco, California, United States of America; 2 Departments of Anatomy and Ophthalmology, University of California San Francisco, San Francisco, California, United States of America; 3 School of Optometry and Vision Science Graduate Group, University of California, Berkeley, California, United States of America; University of San Francisco, United States of America

## Abstract

Keratinizing squamous metaplasia (SQM) of the ocular surface is a blinding consequence of systemic autoimmune disease and there is no cure. Ocular SQM is traditionally viewed as an adaptive tissue response during chronic keratoconjunctivitis sicca (KCS) that provokes pathological keratinization of the corneal epithelium and fibrosis of the corneal stroma. Recently, we established the autoimmune regulator-knockout (Aire KO) mouse as a model of autoimmune KCS and identified an essential role for autoreactive CD4+ T cells in SQM pathogenesis. In subsequent studies, we noted the down-regulation of paired box gene 6 (Pax6) in both human patients with chronic KCS associated with Sjögren’s syndrome and Aire KO mice. Pax6 encodes a pleiotropic transcription factor guiding eye morphogenesis during development. While the postnatal function of Pax6 is largely unknown, we hypothesized that its role in maintaining ocular surface homeostasis was disrupted in the inflamed eye and that loss of Pax6 played a functional role in the initiation and progression of SQM. Adoptive transfer of autoreactive T cells from Aire KO mice to immunodeficient recipients confirmed CD4+ T cells as the principal downstream effectors promoting Pax6 downregulation in Aire KO mice. CD4+ T cells required local signaling via Interleukin-1 receptor (IL-1R1) to provoke Pax6 loss, which prompted a switch from corneal-specific cytokeratin, CK12, to epidermal-specific CK10. The functional role of Pax6 loss in SQM pathogenesis was indicated by the reversal of SQM and restoration of ocular surface homeostasis following forced expression of Pax6 in corneal epithelial cells using adenovirus. Thus, tissue-restricted restoration of Pax6 prevented aberrant epidermal-lineage commitment suggesting adjuvant Pax6 gene therapy may represent a novel therapeutic approach to prevent SQM in patients with chronic inflammatory diseases of the ocular surface.

## Introduction

Squamous metaplasia (SQM) is a devastating, end-stage consequence of many autoimmune diseases that cause aqueous-deficient dry eye, known clinically as keratoconjunctivitis sicca (KCS). Diseases like Steven Johnson syndrome, ocular cicatricial pemphigoid and Sjögren’s syndrome (SS) cause SQM by setting off a multi-step, immune-mediated process that leads to pathological keratinization of the cornea and loss of mucin-secreting goblet cells. Histologically, SQM refers to a process of altered differentiation where the moist, ocular muco-epithelium rich in cytokeratin 12 (CK12) is transformed to a dry, keratinized, “skin-like” epithelium rich in CK10 [1–3]. Despite powerful immunosuppressive and immunomodulatory therapy, autoimmune-mediated dry eye disease is highly recalcitrant to treatment and can progress to corneal opacification and blindness. 

To study the immunopathogenesis of SQM *in vivo*, we characterized a novel murine model of autoimmune-mediated aqueous tear deficiency and SQM [4–6]. Mice deficient in the autoimmune regulator gene (*Aire KO*) spontaneously developed a T cell-mediated exocrinopathy [7,8], which provoked an aqueous deficient dry eye with SQM occurring as early as postnatal week six. Through adoptive transfer studies, we directly implicated CD4+ T cells in the pathogenesis of autoimmune SQM and showed that IL-1/IL-1R1 signaling via resident cell of the ocular mucosal epithelium served as an essential intermediate acting downstream of CD4+ T cells to initiate and perpetuate SQM of the ocular surface [4,9].

Having identified several key steps in the inflammatory pathway that led to SQM, we sought to uncover the specific molecular events that fueled the transition from corneal to epidermal lineage during chronic inflammation. During embryogenesis, the ocular surface epithelium develops from a highly specialized surface ectoderm, via a constellation of induction and regulatory transcription factors. Among these transcription factors, paired-box protein 6 (Pax6), is a master regulator of ocular mucosal lineage commitment that is necessary for normal clonal epithelial growth, activation of limbal stem cells and directing corneal epithelial cell migration [10]. A primary transcriptional function of Pax6 in maintaining corneal lineage homeostasis has been elegantly demonstrated through its transcriptional regulation of corneal-specific CK12 expression in corneal cell lines [11]. Unlike other tissues where Pax6 is lost in the adult, it remains universally expressed throughout the entire differentiated, stratified ocular epithelia of the adult eye (i.e., from cornea, limbus to conjunctiva) [12]. Although the postnatal function of Pax6 in ocular mucosal biology is not fully understood, accumulating evidence shows that under certain pathological conditions, disturbance or breakdown of the corneal epithelium is consistent with a change in Pax6 expression. A decline of ocular surface Pax6 expression has been observed in a number of ocular surface diseases, ranging from immune disorders like Steven’s Johnson syndrome, to genetic defects, like aniridia [13]. Ocular SQM represents a common tissue response that occurs during these disease processes, where CK12 to CK10 transition occurs [3]. Yet, the association between Pax6 loss and SQM development in ocular surface disease does not define the potential functional significance of this relationship in upsetting the fundamental tissue phenotype. In view of this, elucidating the postnatal regulatory role of Pax6 in the cellular mechanisms of corneal lineage commitment in health and disease would advance our understanding of the mechanisms by which this master regulator shapes adult tissues, as well as disruption of this process in the absence of Pax6. 

In the current study, we explored the immunopathogenesis of aberrant ocular mucosal lineage commitment in the setting of autoimmunity. We revealed Pax6 loss from mucosal epithelial cells lining the ocular surface of both Aire KO mice and human patients with chronic KCS . Loss of Pax6 occurred as a consequence of autoreactive CD4+ T cell infiltration and activation of IL-1R1 on resident cells of the ocular surface. Through forced expression of Pax6 *in vivo* we restored the ocular mucosal phenotype in Aire KO mice, thus, supporting the functional association between Pax6 dysregulation and SQM development. The clinical implications of maintaining or restoring ocular mucosal Pax6 to prevent the blinding consequences of SQM are discussed. 

## Materials and Methods

All materials were purchased from Sigma (St. Louis, MO, USA), except: defined keratinocyte serum free medium (KSFM, Gibco-BRL, Grand Island, NY), Dispase II (Roche, Indianapolis, IN), DAB substrate (Vector Laboratories, Burlingame, CA), and DAPI (4',6'-diamino-2-phenylindole) (Molecular probes, Eugene, OR). Purified rat anti-mouse CD4 antibody was from BD Biosciences (San Jose, CA). Pax6 rabbit polyclonal antibody, Cytokeratin 10 and Cytokeratin 12 goat polyclonal antibodies were from Santa Cruz Biotechnology (Santa Cruz, CA). Horseradish peroxidase (HRP)-conjugated goat-anti-mouse secondary antibody was from Jackson ImmunoResearch Laboratories (West Grove, PA). Alexa Fluor 488 goat anti-mouse, Alexa Fluor 488 donkey anti-goat, Alexa Fluor 594 goat anti-rabbit secondary antibodies were from Invitrogen (Grand Island, NY).

### Human Subject Recruitment And Sample Collection

All aspects of the human subject studies presented in this manuscript were approved by the UCSF Committee for Human Research (Approval number: 10-04144). Fifteen patients with KCS (11 with SSand 4 with non-SS KCS) and seven healthy controls were recruited from the University of California San Francisco (UCSF) Oral Medicine and Proctor Foundation clinics. Informed consent was obtained in writing from all subjects prior to participation. Subjects were presented with a written document describing the study objectives and sample collection procedures. A quantitative ocular staining score (OSS) developed by the Sjögren’s International Clinical Collaborative Alliance (SICCA) was employed to assess the severity of KCS epitheliopathy [14]. The OSS ranges from 0 to 12. A score of “0” indicates no corneal or conjunctival staining with fluorescein or lissamine green dye, respectively. A score of 12 indicates severe staining of the cornea and conjunctiva with confluent fluorescein staining in the pupillary area and the presence of corneal filaments. An OSS ≥ 4 was considered positive for KCS and inclusion in the study. Patients with SS had KCS and either (1) labial salivary gland biopsy showing focal lymphocytic sialadenitis (focus score > 1); or (2) positive serological results for anti-SS-A/Ro or SS-B/La antibody.. Exclusion criteria included known diagnosis of HCV, HIV, sarcoidosis, amyloidosis, GVHD, systemic lupus erythema, rheumatic arthritis or preexisting lymphoma. Impression cytology was performed to harvest superficial conjunctival epithelial cells for RNA extraction. Total RNA was extracted from tissues using Qiagen RNeasy Micro or Pico Kit. RNA was reverse transcribed using a high capacity cDNA Reverse Transcription Kit from Applied Biosystems. The TaqMan ABI 7500 system from Applied Biosystems was used to monitor Pax6, SPRR1B, IL-1β and cytokeratin gene expression. 

### Animal Model

Mice were handled in strict accordance to UCSF animal welfare guidelines for animal care. The protocol was approved by the Institutional Animal Care and Use Committee at UCSF (Approval number: AN089075-02). Aire-deficient mice were generated by targeted disruption of the murine *Aire* gene (OMIM 240300) as previously described [15]. Aire-deficient mice were backcrossed onto the non-obese diabetic (NOD) Lt/J background for more than 10 generations and then crossed with NOD mice deficient in functional IL-1 receptor type 1 (point mutation in *IL-1R1* loci, OMIM 147810) purchased from Jackson Laboratory (Bar Harbor, ME) to create NOD.*Aire* ^-/-^IL-1R1^-/-^ mice. In addition, severe combined immune deficiency (scid) mice (NOD.CB17-*Prkdcscid*/J strain, Jackson Laboratory, Sacramento, CA) were crossed to NOD.*IL-1R1* ^-/-^ to generate IL-1R1^-/-^ scid mice on NOD background. Genomic DNA isolated from tail clippings was genotyped for the *Aire*, *IL-1R1*, and *scid* mutations by PCR with manufacturer recommended specific primers and their optimized PCR protocols. All the mice were 7-8 weeks of age when sacrificed.

### Adoptive Transfer Procedure

Lymphocytes from four cervical lymph nodes and spleens of wild type (WT), Aire KO and Aire KO mice lacking functional IL-1R1 (all on the NOD background) were used for adoptive transfer studies. The CD4^+^ T cell population was enriched by magnetic bead sorting and the purity was confirmed by flow cytometry, as previously described [9]. CD4^+^ T cell-enriched lymphocytes in 100 μl PBS (5x10^6^ cells/mouse) were injected to the tail vein of NOD.scid mice recipients either IL-1R1 sufficient (IL-1R1^+/+^ scid) or deficient (IL-1R1^-/-^ scid). 

### Isolation of Murine Corneolimbal Epithelial Sheets

Mouse corneolimbal sheets were isolated as previously reported [16]. One eye per mouse was enucleated by forceps, washed in PBS, and digested by Dispase II (10mg/ml) in KSFM at 4°C for 18 hours. Using jewelers forceps, an intact corneolimbal epithelial sheet was surgically peeled off each eye and used for RNA isolation. A minimum of three mice from each genotype was used for each experiment.

### Transcriptional Profiling of Pax6, IL-1β, and Cytokeratins using Taqman PCR

Corneolimbal sheets were isolated from enucleated eyes, with a modification of our previously described protocol [17]. In brief, the whole corneolimbal sheet was surgically peeled off with jeweler’s forceps from the underlying stroma of enucleated eyes, after overnight enzymatic digestion of the epithelial basement membrane by Dispase II (10mg/ml) in serum-free keratinocyte medium (Gibco) at 4°C. Total RNA was extracted from those cell sheets using an RNeasy Mini RNA isolation kit (Qiagen, Germany). Total RNA was eluted from mini columns with 30μl of RNase-free water. Starting with 1μg of RNA, 50μl cDNA was synthesized by using TaqMan Reverse Transcription Reagents, which contains Oligo d(T)16 and random hexamer primers, RNase inhibitor, deoxyNTPs mixture and MultiScribe reverse transcriptase. The reaction was performed for 10 min at 25°C, 15 min at 42°C, and 5 min at 95°C. cDNA was stored at -20°C until use. To compare the relative abundance of Pax6, CK12, CK10, CK14, IL-1β and SPRR1B transcripts, we used a TaqMan Probe fluorogenic 5´nuclease chemistry-based gene expression assay with exon boundary-crossing primers (gene assay ID: 00443081_m1, 00839769_m1, 03009921_m1, 00516876_m1, 00434228_m1, 02016340_s1, respectively). One microliter of cDNA was used in the 20μl reaction mix in each well of the 96-well plate. Real-time PCR was performed using thermal cycling conditions at 95°C for 10 min, followed by 40 cycles of 15 sec at 95°C and 1 min at 60°C for amplification in the ABI Prism 7500 Real Time PCR System (Applied Biosystems, Fort City, CA). All assays were performed and compared in four technical replicates to a housekeeping gene, GAPDH (gene assay ID: 03302249_g1). GAPDH was chosen as the reference gene based on published work demonstrating that its expression is consistently maintained in inflamed corneal tissues [18–20], as well as in our own studies of Aire KO mice following various interventions [4,6,21]. C_t_ data were derived from 3-7 mice in each group. The amplification efficiency of primers was obtained by constructing a standard curve for each primer. The relative quantity (RQ) of gene expression was calculated using the Pfaffl method as previously described [22]. Positive and negative quality controls for reproducibility, reverse transcription and genomic DNA contamination were assessed and found to be acceptable. 

### Immunostaining, Immunofluorescence and Histopathology

Eyes were embedded in OCT and sectioned into 7µm thick sections. Sections were fixed with cold acetone for 10 minutes at -20°C prior to staining. Antibodies directed against Pax6, CK12, and CK10 were used to establish Pax6 tissue distribution, cytokeratin expression profile and cornified envelope formation. For immunofluorescent labeling of CK10 and CK12, sections were blocked with 5% normal horse serum for 1 hour at room temperature followed by overnight incubation in primary antibody at 4°C. All cytokeratin primary antibodies were used at a 1:100 dilution. Alexa Fluor secondary antibodies were used at 1:400 dilution, followed by 5-minute incubation with DAPI and mounted with Fluorsave. Negative controls included the omission of the primary antibody. Histopathology of the ocular tissues was examined by hematoxylin & eosin (H&E) staining. All staining procedures were carried out according to previously optimized protocols for Aire KO mice established in our laboratory [5].

### Cytospin Assay

Protein expression of Pax6, CK12 and CK10 were examined at the single-cell level by cytospin assay. Isolated corneolimbal sheets were trypsinized to single cell suspension. A 10% solution of FBS was added to stop trysinization and cells were resuspended in 100μl KSFM. From the cell suspension, 50 μl was added to a slide chamber and spun at 1000 rpm for 8 minutes using the StatSpin Cytofuge. Slides were allowed to air dry then fixed with 95% alcohol prior to staining. Quantitative data was generated by manually counting the number of cells expressing specific markers from multiple view-fields of the cytospin staining from different mice and representing it as a percentage of cells. 

### Forced Expression of Pax6 using Adenovirus

Adenoviral vectors carrying Tet-CMV-EGFP-Pax6 construct Pax6 (Ad-EGFP-Pax6) or empty vector expressing EGFP only (Ad-EGFP) were provided by Dr. Chia-Yang Liu (University of Cincinnati, College of Medicine). Methods used to construct these recombinant adenoviruses have been described previously [23]. Bilateral sub-conjunctival injection of Aire KO mice with 10μl of 1x10^7^ pfu/ml Adeno-EGFP-Pax6 or Adeno-EGFP vector viral preparation was performed on Aire KOs and WTs at 6 weeks of age (n=5 mice per group). The mice were fed with sweetened 1mg/ml Doxycycline water containing 20% sucrose for 5 days following injections. Ocular integrity was evaluated using lissamine green dye before and 5 days following injection. Mice were euthanized at day 5-post injection. Eyes were enucleated and snap-frozen in OCT for transfection verification, histological analysis and phenotypic characterization.

### Statistics

To compare qPCR gene expression and quantitative cytospin data between WT and Aire KO mice, we used the unpaired Student’s t-test or the Kruskal Wallis test depending on whether the data was normally distributed We adjusted for multiple comparisons using the Bonferroni correction. We modeled the relationships between Pax6 and readouts of SQM, using linear regression. All analyses were done using the statistical software Stata 9.0 (College Station, TX) for Macintosh.

## Results

### Clinical and histological alteration of the ocular mucosal epithelium during chronic, autoimmune-mediated squamous metaplasia

**Figure 1:**
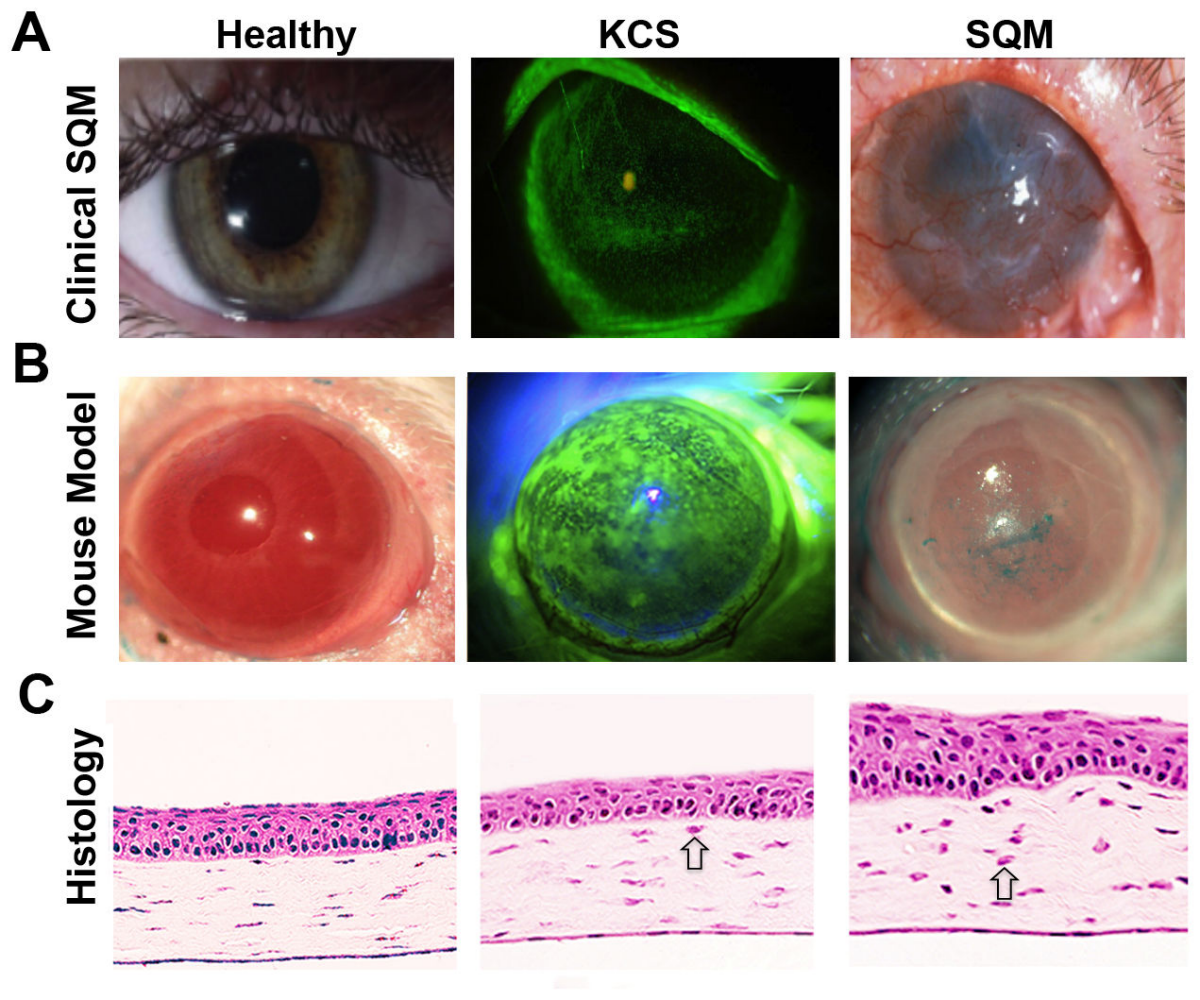
Keratoconjunctivitis sicca (KCS) and squamous metaplasia (SQM) of the ocular surface in response to CD4+ T cell-mediated autoimmunity. Representative images of autoimmune-mediated ocular surface disease in a (**A**) human patient and (**B**) Aire KO mouse. Aqueous tear deficiency leads to KCS with loss of epithelial integrity indicated by punctate fluorescein staining (A&B-middle panels). SQM is accompanied by pathological keratinization, corneal opacification and vascularization (A&B - right panels). (**C**) H&E staining of cryosectioned eyes from Aire KO mice reveals representative histological changes associated with disease progression. Open arrows indicate infiltrating immune cells in the corneal stroma.

The cornea is the major refracting surface of the eye where the absence of blood vessels and regular arrangement of stromal collagen fibrils is essential to maintaining corneal transparency (Figure 1A, left). SQM is a devastating consequence of aqueous deficient dry eye, also known as keratoconjunctivitis sicca (KCS) (Figure 1A, middle panel) where pathological keratinization couples with subepithelial fibrosis to cause corneal opacification and blindness (Figure 1A, far right). While CD4+ T cell-mediated inflammation has been implicated in the pathogenesis of KCS and the development of SQM, little is known about the immunopathogenic mechanisms and effective treatments are severely lacking. 

As a model to mimic the clinical features of SQM in human patients, Aire KO mice spontaneously develop CD4+ T cell-mediated autoimmune disease that provokes severe KCS (Figure 1B, middle) with progressive keratinization and corneal opacification (Figure 1B, right) noted by 8 - 10wks of age [6,9]. Histopathology of the Aire KO mouse cornea undergoing SQM demonstrates persistent immune cell infiltration (indicated by open arrows) enlargement of superficial cells and morphological disorganization of corneal epithelial cells where basal cells form heterogeneous basal/parabasal clusters (Figure 1C, middle). In severe disease, corneal cells take on skin-like characteristics with increased cellular stratification, disorganization and pathological keratinization (Figure 1C, right) [4,24]. 

### Master regulator of corneal phenotype, Pax6, is downregulated in corneal epithelial cells undergoing SQM

**Figure 2:**
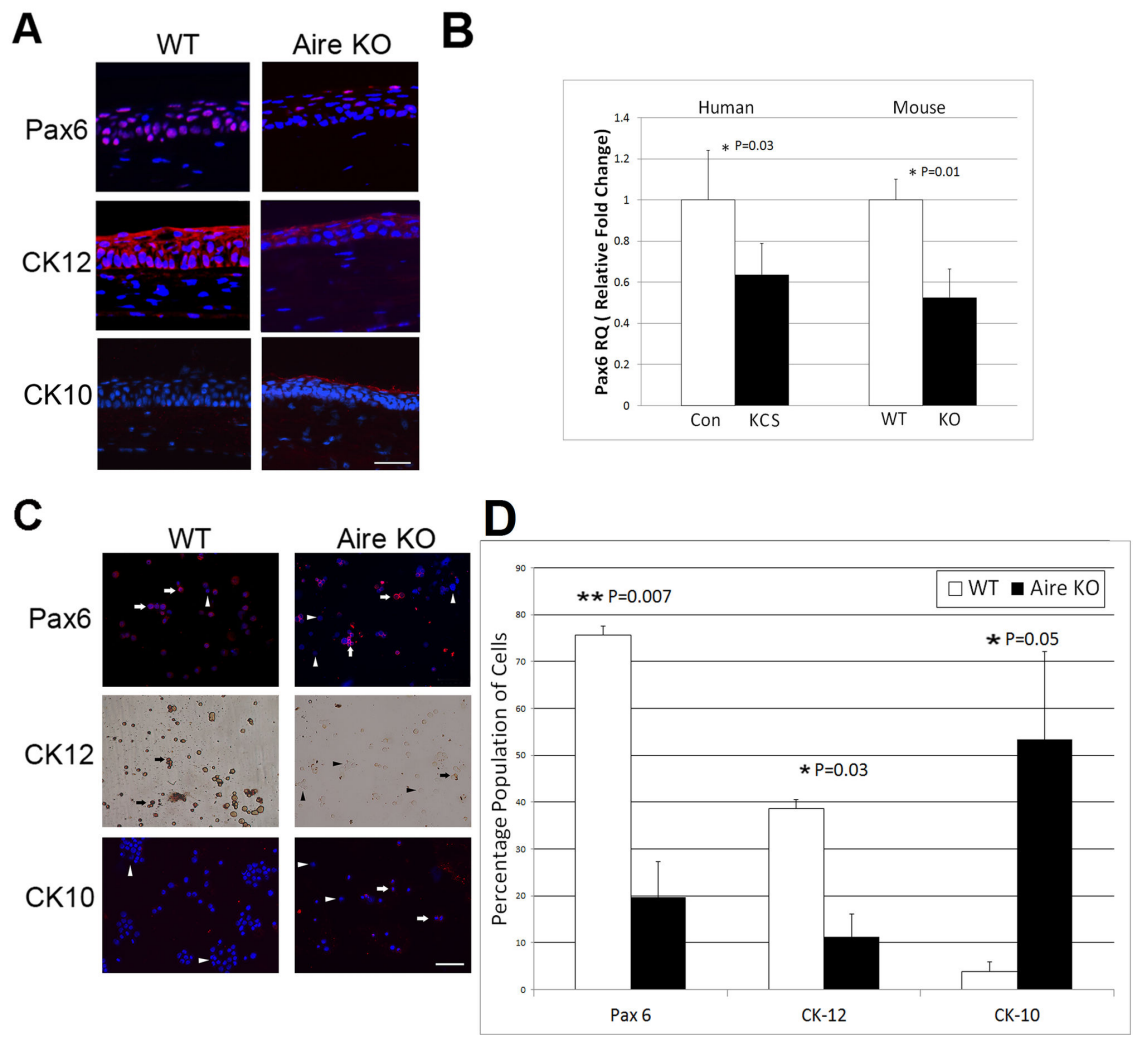
Altered lineage commitment of the ocular mucosal epithelium in autoimmune-mediated KCS/SQM. (**A**) Immunofluorescent staining of nuclear Pax6 (red, top), corneal-specific CK12 (red, middle) and epidermal-specific CK10 (red, bottom) in the corneal epithelium of WT and Aire KO mice. Scale bar = 50μm (**B**) TaqMan qPCR analysis of Pax6 gene using corneolimbal epithelial cells from WT and Aire KO mice and impression cytology specimens from healthy human control (Con) and chronic KCS (KCS) patients. Pax6 expression in WT mice or healthy control patients was used as the reference (designated 1-fold) to generate relative quantitation (RQ) values. Data are shown as mean RQ±SE. Seven mice were studied per group, as well 15 KCS patients and 7 healthy controls. Unpaired T-test (mouse) or Wilcoxan Rank Sum test (human) was used to test for differences between groups, with P<0.05 (*) and P<0.01 (**) considered statistically significant. (**C**) Cytospin was used to assess single-cell expression of Pax6 (red, upper panels), CK12 (brown, middle panels) and epidermal cytokeratin K10 (red, bottom panels) in WT and KO mice. Arrows and arrowheads indicate positive and negative stained cells, respectively. (**D**) Quantitative analysis of cytospin specimens expressed as the percentage of cells staining positive for Pax6, CK12 and CK10. Data were generated by analyzing three separate fields of view from an individual specimen with a minimum of three animals analyzed per group. Group mean comparisons were assessed by T-test, with P<0.05 (*) and P<0.01 (**) considered statistically significant.

The specific regulatory events that provoke SQM and cornified envelope formation in autoimmune KCS are unclear. With Pax6 serving as the master transcription factor that governs corneal differentiation, we examined Pax6 expression in Aire KO mice and human patients with SS. In WT mice, Pax6 was ubiquitously expressed in all layers of corneal epithelium, including basal cuboidal cells, suprabasal winged cells, and superficial squamoid cells. Accordingly, CK12 expression was homogenous throughout the stratified corneal epithelium while epidermal-specific cytokeratin 10 was undetectable (Fig. 2A, left panels). In contrast, Pax6 and CK12 expression were attenuated in the corneas of Aire KO mice. The distribution of CK12 was patchy and discontinuous throughout the full thickness of the stratified epithelium, where the majority of cells demonstrated negative immunoreactivity to Pax6 and positive immunoreactivity to CK10 (Fig. 2A, right panels). Changes in Pax6 were also noted at the transcript levels (Fig. 2B). Using TaqMan qPCR, we found reduced ocular surface Pax6 expression in both Aire KO mice and human patients with KCS. Pax6 was reduced ~ 2-fold in corneolimbal epithelial cells isolated from Aire KO mice with a mean±SE RQ value of 0.52±0.13 in Aire KO vs.1.0±0.10 in WT; P=0.01. Expression was similarly reduced in human patients with KCS vs. healthy controls using desquamated cells isolated from the bulbar conjunctiva by impression cytology (0.63±0.15 in KCS vs. 1.0±0.24 in Controls; P=0.03) (Figure 2B). Analysis of corneolimbal epithelial cells expressing Pax6, CK12 and CK10 by cytospin revealed decreased Pax6 and CK12 protein at the single-cell level with a coordinated increase in CK10 (Figure 2C). The percentage of corneal cells expressing Pax6 in Aire KO mice was significantly reduced with respect to the wild type (19.7±7.6 in Aire KO vs. 75.6±11.1 in WT; P=0.007). Likewise, the percentage of CK12 expressing cells was significantly reduced (11.2±4.9 in KO vs. 38.6±9.2 in WT; P=0.03), and the percentage of CK10 expressing cells was significantly increased (53.3±18.7 in KO vs. 3.9±1.9 in WT; P=0.05) Figure 2D. 

### Loss of Pax6 is mediated via the infiltration of autoreactive CD4+ T cells and local signaling via IL-1R1

**Figure 3:**
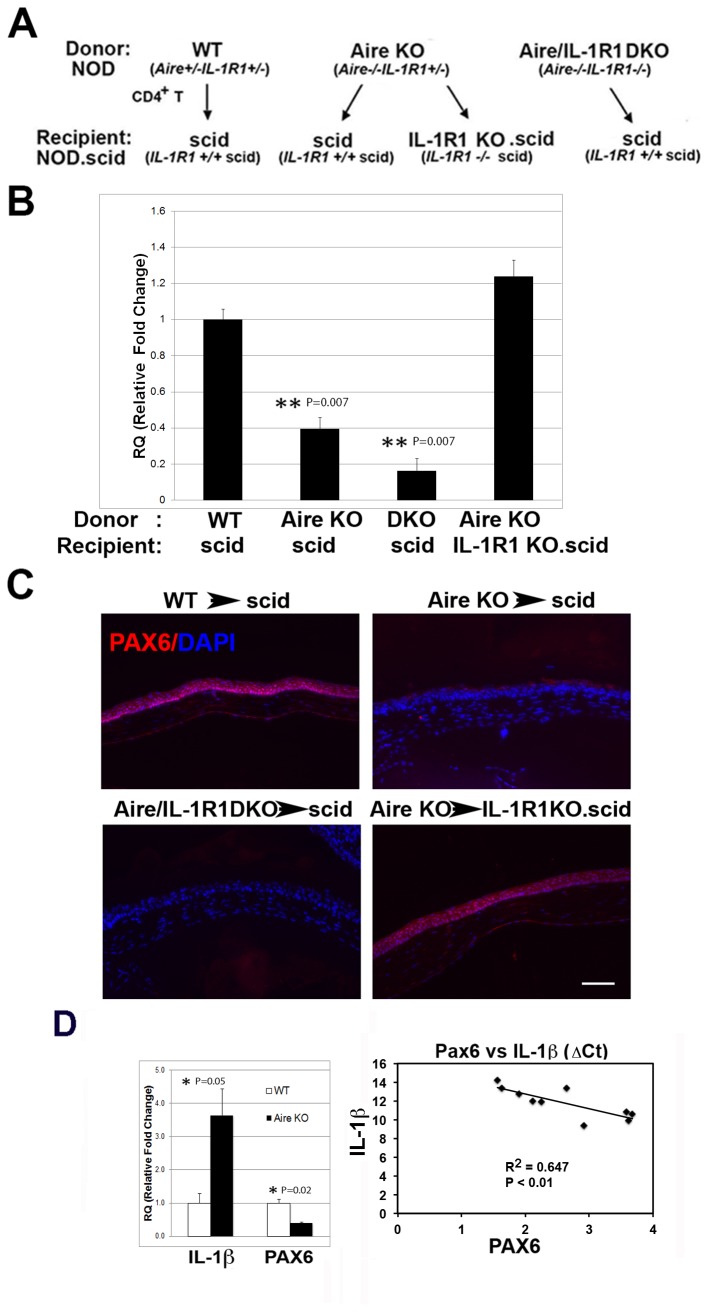
Role of IL-1R1/IL-1 signaling in mediating Pax6 loss and altered lineage commitment in autoimmune-mediated KCS/SQM. (**A**) Adoptive transfer (AT) experimental design. (**B**) Transcriptional profile of corneolimbal epithelial Pax6 in the four AT groups with control group (WT scid) set as the reference (designated 1-fold) to generate relative quantitation (RQ) values of Pax6 expression. Data are shown as mean RQ value ± SE; statistically significant differences are noted as P<0.01 (**); n=5 per group. (**C**) Immunolocalization studies of Pax6 staining in the four AT groups (red); Scale bar = 50μm. (**D**) IL-1β and Pax6 transcript levels in WT and Aire KO mice expressed as mean ± SE RQ values where an arbitrary WT control mouse was set as the reference (designated 1-fold); P<0.05 (*) indicates statistical significance; n = 3 per group. Regression analysis of transcript levels indicates Pax6 and IL-1β expression are negatively correlated in Aire KO mice.

Previously, we identified CD4+ T cells as the primary effector cell promoting KCS and SQM in Aire KO mice [8,9]. Notably, the detrimental effects of autoreactive CD4+ T cells on ocular surface integrity and induction of pathological keratinization required the activation of local signaling via IL-1R1 [4,6]. Using an adoptive transfer paradigm, we compared Pax6 expression in immunodeficient recipients of CD4+ T cells from Aire KO or WT control mice in the presence and absence of locally expressed IL-1R1. As summarized in Figure 3A, CD4^+^ T cells from three different donor phenotypes, WT (*Aire* ^*+/+*^ *IL-1R1* ^*+/+*^), Aire KO (*Aire* ^*-/-*^ *IL-1R1* ^*+/+*^) and Aire/IL-1R1 DKO (*Aire* ^*-/-*^ *IL-1R1* ^*-/-*^), were transferred to immunodeficient recipients either sufficient (*IL-1R1^+/+^.scid*) or deficient (*IL-1R1^-/-^.scid*) in IL-1R1. With this approach we were able to differentiate whether the direct target of IL-1 signaling was pathogenic CD4^+^ T cells or local resident cells of the ocular surface. Immunodeficient recipients of CD4^+^ T cells from WT (WT → scid) and Aire KO mice (Aire KO → scid) served as negative and positive controls, respectively. Pax6 expression in the cornea was assessed by qPCR (Figure 3B) and immunofluorescence (Figure 3C). To calculate Pax6 mRNA expression, results from control mice (WT → scid) were designated 1-fold to generate relative quantitation (RQ) values for the remaining three groups, with Aire KO → scid = 0.395±0.06; Aire/IL-1R1 DKO → scid = 0.163±0.06; and Aire KO→IL-1R1KO.scid = 1.23±0.08. Notably, scid recipients of autoreactive CD4+ T cells from Aire KO and Aire/IL-1R1 DKO mice showed a 2.73-fold (P=0.007) and 8.92-fold (P=0.007) decrease in ocular surface Pax6 transcript relative to controls. The reduction in Pax6 induced by autoreactive CD4+ T cells occurred only when immunodeficient recipients expressed IL-1R1. Pax6 transcript was maintained in IL-1R1-deficient scid mice following injection of autoreactive CD4+ T cells (Aire KO → IL-1R1 KO.scid) at a level equivalent to that of controls (WT → scid) (1.22±0.12 vs. 1.0±0.11, p>0.05). Similar results were noted at the protein level (Figure 3C) where nuclear Pax6 persisted throughout the mucosal epithelium of Aire KO → IL-1R1 KO.scid mice, compared to nearly complete loss of ocular Pax6 in Aire KO → scid mice where local IL-1R1 was intact. Thus, loss of master regulator, Pax6, in corneal epithelial cells undergoing SQM was dependent on CD4+ T cell infiltration and local activation of IL-1R1 on resident cells of the ocular tissues. Accordingly, we noted a ~ 3.5-fold increase of IL-1β transcript in corneolimbal cells isolated from the eyes of Aire KO vs. WT mice (3.64±0.79 vs. 1.0±0.29, P=0.05), that coincided with a ~ 2.5-fold decrease in Pax6 mRNA (0.407±0.01 vs. 1.0±0.10, P=0.02). Linear regression analysis confirmed a significant correlation between loss of Pax6 and increased expression of IL-1β (R^2^ = 0.647, p < 0.01 (Figure 3D)). 

### Downregulation of Pax6 predicts the cytokeratin maturation profile of corneolimbal epithelial cells during chronic inflammation

**Figure 4:**
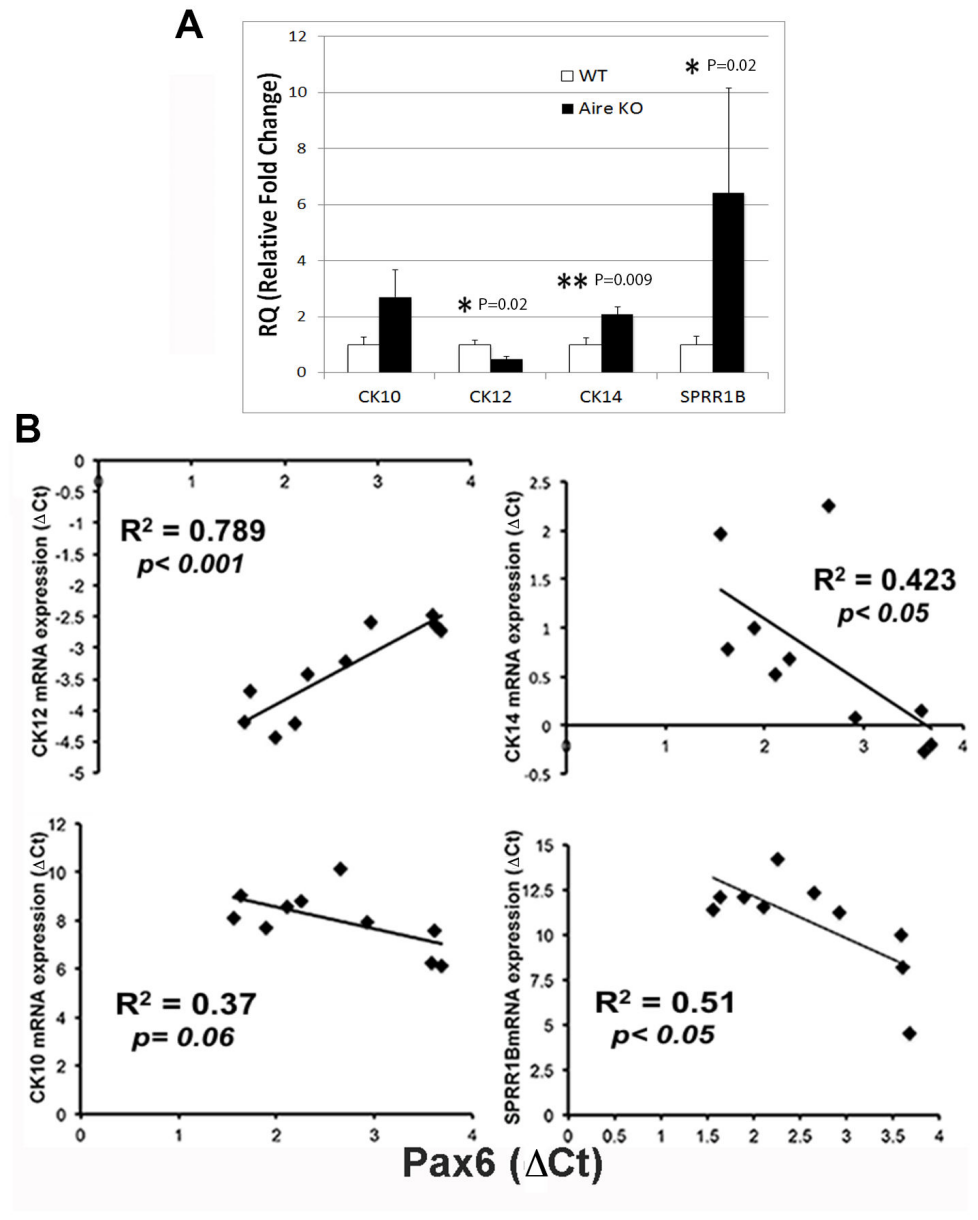
Cytokeratin expression profile of the corneolimbal epithelium in autoimmune-mediated KCS/SQM. (A) Transcriptional profiling of ocular surface cytokeratin expression in corneolimbal epithelial cells isolated from WT and Aire KO mice. Quantitative PCR for CK12, CK10, SPRR1B and CK14 were expressed as relative quantitation (RQ) values where an arbitrary WT control mouse was set as the reference (designated 1-fold). RQ values are shown as mean ± SE; n=3 mice per group. Unpaired T-test (CK12 and CK14) or the Kruskal-Wallis Rank Sum test (SPRR1B and CK10) was used to test for differences between WT vs. Aire KO with P<0.05 (*) and P<0.01 (**) considered statistically significant. (**B**) Downregulation of Pax6 predicts the cytokeratin maturation profile of corneolimbal epithelial cells. Regression analysis shows Pax6 was positively correlated with CK12 but negatively correlated with CK10, CK14 and SPRR1B. R^2^ values are provided.

Next, we explored the cytokeratin expression profile of the ocular mucosal epithelium undergoing SQM. As ocular surface disease progressed, decreased levels of CK12 transcript were paralleled by increased expression of epidermal marker, CK10, keratin envelope protein, SPRR1B, and stratified squamous basal cell marker, CK14 (Figure 4A). CK12 gene expression was reduced ~ 2-fold from an RQ (relative quantity) of 0.479±0.09 in Aire KO mice vs. 1.0±0.16 in WT mice; P=0.02, CK10 was increased ~ 2.7-fold (2.69+0.98 Aire KO mice vs. 1.0±0.11 in WT mice P>0.05) and SPRR1B was increased ~6.5-fold (6.41±3.7 Aire KO mice vs. 1.0±0.29 in WT mice; P=0.02). Notably, in the most severe cases of end-stage SQM, complete loss of Pax6 produced a phenotype where CK14+ cells were distributed throughout the corneal epithelium, leading to ~ 2-fold increase in CK14 compared to WT mice (2.08±0.26 Aire KO mice vs. 1.0±0.23 in WT mice; P=0.009). The potential role of Pax6 downregulation as a disease sentinel for SQM was highlighted in Figure 4B, where regression analysis indicated Pax6 loss was a predictor of decreased expression of cytokeratin CK12 (R^2^=0.789, P<0.001) and increased expression of CK10 (R^2^=0.37, P=0.06), SPRR1B (R^2^=0.51, P<0.05), and CK14 (R^2^=0.423, P<0.05). 

### Forced expression of Pax6 adenovirus restores corneal phenotype in Aire KO mice

**Figure 5:**
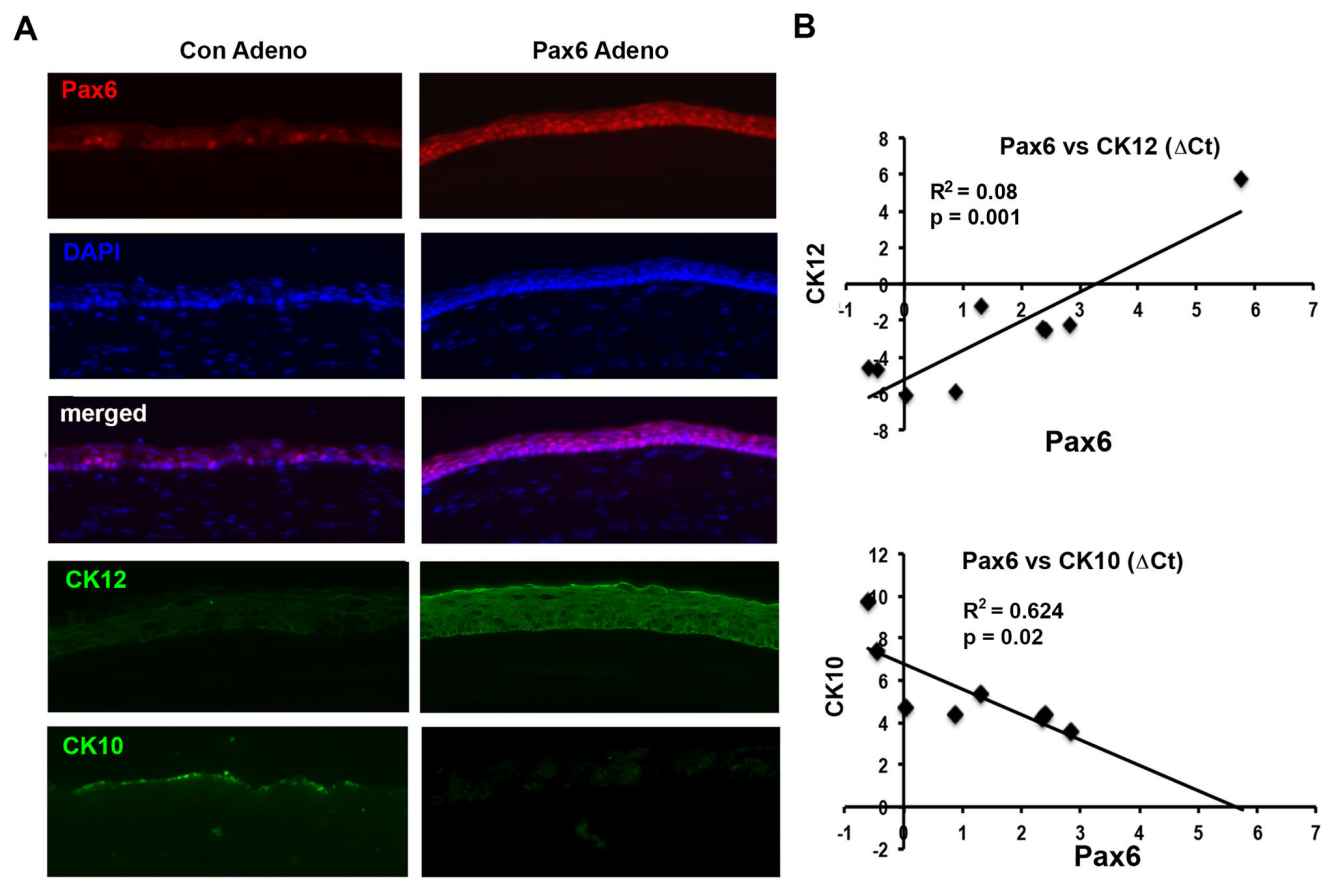
Pax6 adenovirus restores corneal phenotype in autoimmune-mediated KCS/SQM. (**A**) Immunostaining of corneal Pax6 (red), CK12 (green), and CK10 (green) in Aire KO mice 5 days after injection with Pax6-adenovirus (Pax6-Adeno) or control adenovirus (Con-Adeno). Images are representative of three (Con-Adeno) and five (Pax6-Adeno) mice per group. Nuclei is stained blue with DAPI. (**B**) Linear regression indicates cytokeratin switch from CK12 (top) to CK10 (bottom) was highly correlated to recovery of Pax6 following forced expression.

To provide a functional link between Pax6 loss and aberrant lineage commitment of corneolimbal epithelial cells during chronic inflammation, we tested the effect of adenoviral vector-transferred Pax6 in re-directing epithelial progenitors in keratinized ocular surface back to mucosal phenotypes. After demonstrating the safety and efficacy of Pax6 adenoviral gene transfer *in vivo*, we injected 5 X 10^7^ PFU (in 10 μl) of EGFR-tagged adenoviral vector carrying Pax6 or empty vector into the subconjunctival space of 6 wk-old mice (Figure 5A). Aire KO mice received Adeno/Pax6 (n=5) or Ad/control vector (n=3). Mice were sacrificed 5 days later and eyes embedded in OCT for immunostaining. After 5 days, the majority of ocular surface epithelial cells expressed Pax6 in the nucleus. Immunostaining revealed a coordinated increase in CK12 and decrease in CK10 staining of the corneal epithelium following forced expression of Pax6. Recovery of Pax6 mRNA transcript levels following adenoviral injection was highly correlated to increased expression of cytokeratin CK12 (R^2^=0.80, P=0.001) and decreased expression of CK10 (R^2^=0.624, P=0.02) (Figure 5B). These studies provided direct evidence supporting the functional role for Pax6 in maintaining corneal phenotype in adult mice, while loss of Pax6 during chronic inflammation permits aberrant differentiation to an epidermal lineage. Restoration of corneal lineage upon reintroduction of Pax6 adenovirus revealed a novel therapeutic role for Pax6 in managing the devastating consequences of SQM.

## Discussion

 We sought to decipher the mechanism whereby autoimmune-mediated inflammation promotes vision threatening SQM with the intent to develop early diagnostic tools and targeted therapies to prevent corneal blindness. Using Aire KO mice as a model of SQM, we showed that the principle immune effectors, CD4+ T cells, and local signaling via IL-1R1 provoked aberrant lineage commitment of the ocular mucosa through the loss of master transcriptional regulator, Pax6. Based on Pax6’s essential role for prenatal oculogenesis and postnatal corneal phenotype homeostasis, we hypothesized that loss of Pax6 provided the cellular and molecular link between systemic autoimmunity and the genesis of ocular surface disease.

 Often following injury, post-natal tissue adopts prenatal embryonic differentiation pathways to repair the injury and thus, maintain the tissue-specific differentiation hallmarks. However, in certain pathological conditions, an alternative differentiation code is adopted to better tackle the environmental stress [25]. Numerous prenatal animal studies have demonstrated that Pax6 is the earliest transcription factor dictating commitment to ocular epithelial lineage in eye morphogenesis [13]. While Pax6 is retained throughout the entire differentiated stratified ocular epithelia of the adult eye (i.e., from cornea, limbus to conjunctiva) [12], the postnatal role of Pax6 has been largely undefined. In aqueous tear deficiency, persistent desiccating stress sets off a protective response that adopts an epidermal-like phenotype, with the laying down of a keratinized envelope. Here, we provide the first direct *in vivo* evidence demonstrating a functional connection between Pax6 loss and SQM development in the setting of chronic inflammation. 

 By definition, metaplasia implies the replacement of one differentiated cell type with another mature differentiated cell type. The ocular surface epithelium shares many of the basic features of the epidermal system, including a stratified squamous phenotype and expression of universal and tissue-specific cytokeratins. In the epidermis, differentiation involves a switch of cytokeratin expression from the universal stratified epithelial cytokeratin pair, CK5/CK14 to epidermis-specific CK1/CK10 [26,27], or corneal-specific CK3/CK12 [28]. Phenotypic differences between skin and cornea occur in the late stages of differentiation when cornified envelope proteins form the keratinized epidermis. While keratinization clearly distinguishes the epidermal and corneal phenotypes, it is important to note that the ocular mucosal epithelium expresses all the proteins needed to synthesize a cornified envelope. Our studies suggested that in the setting of chronic inflammation, genes essential to the production and crosslinking of a keratinized epithelium became activated in the absence of Pax6 and provoked pathological keratinization. 

Previously, we established keratin envelope protein, SPRR1B, as a biomarker upregulated in SS patients and Aire KO mice that predicted the presence of ocular epithelial staining and expression of IL-1β [6,9]. Yet, as part of the keratin envelope, SPRR1B was detected only after keratinization had occurred. Here, we provide evidence that Pax6 downregulation in the ocular surface epithelium serves as a disease sentinel for IL-1-mediated SQM. The specific mechanism whereby IL-1R1 activation provokes Pax6 loss and altered lineage commitment is unknown and requires further exploration. The direct association between Pax6 downregulation and inflammation has been noted in the regulation of insulin promoter activity in pancreatic beta cell lines where decreased Pax6 mRNA expression occurred following treatment with inflammatory cytokines, including IL-1β [29]. In the oral mucosa, IL-6 mediated inflammation promotes squamous cell carcinoma through the hypermethylation of Pax6, suggesting epigenetic gene silencing may be an important consequence of chronic inflammation during tumorigenesis [30]. Pax6 also functions in the maintenance and repair of the adult corneal epithelium through transcriptional regulation of matrix metalloproteinase, MMP-9. Interestingly, mice heterozygous for Pax6 demonstrated reduced MMP-9 expression and increased levels of inflammation in a model of epithelial injury. Thus, the downstream consequence of pro-inflammatory cytokine release in response to cell injury may provoke the aberrant regulation of corneal epithelial maintenance and repair through loss of Pax6 and the deactivation of MMP-9 [31]. 

 While in the majority of Aire KO mice we noted a shift from CK12 to CK10 characteristic of SQM, in the complete absence of Pax6, we noted an atypical variant of SQM where CK14 was expressed throughout the full corneal thickness and neither CK12+ nor CK10+ terminally differentiated cells were observed (data not shown). The significance of this observation requires further investigation, but may suggest complete loss of corneal lineage guardian, Pax6, provokes the de-differentiation of basal progenitor cells to an anaplastic state. Notably, loss of CK12 and CK10 expression has been noted in epithelial clones generated from Pax6-negative pannus tissue removed from the eyes of human patients with SQM resulting from aniridia and Stevens-Johnson syndrome [13]. It has been shown that signals from the underlying niche are essential in the tissue-specific regulation of Pax6 expression. Thus, we are currently examining the hypothesis that during homeostasis, Pax6 regulates progenitor cell proliferation to maintain ocular surface-specific lineage commitment. During chronic inflammation, lymphocytic infiltration of the limbal niche and the release of IL-1β provoke Pax6 loss, thereby releasing the control and permitting the onset of aberrant differentiation or the persistence of an undifferentiated state [32–35].

 The functional link between Pax6 loss and SQM development suggested that preventing Pax6 reduction or reinstituting Pax6 function could retain/restore mucosal phenotype. Accordingly, restoration of Pax6 using adenoviral gene transfer prevented aberrant epidermal-lineage commitment in Aire KO mice. While to our knowledge, we are the first to restore corneal lineage *in vivo* using Pax6 gene transfer, a similar approach has been explored in studies of neurogenic cell fate in the forebrain subventricular zone of neonatal mammals where over-expression of Pax6 through *in vivo* retro- or lenti-viral injections was sufficient to promote neuronal lineage development [27]. The biological significance and therapeutic implications of Pax6’s postnatal regulation on homeostatic corneal progenitors will help our quest for a breakthrough in the diagnosis, prevention and treatment of SQM. Our results suggest that adjuvant gene therapy with Pax6 may compliment anti-inflammatory agents as a novel therapeutic approach to prevent SQM in autoimmune disorders of the ocular surface. Given the events related to lineage determination of the ocular surface epithelia are poorly defined, the influence of chronic inflammation represents an exciting new area of research in corneal development where the strategic use of inflammatory disease models, such as the Aire KO mouse, provides an opportunity to explore the molecular events that dictate lineage commitment during the normal and diseased states. 
